# Supplementary material for: Evaluating Prescription Pattern and Effectiveness of Antihypertensive Drugs in Non-Operated Aortic Dissection Patients
Source: J Clin Med. 2023 Mar 1;12(5):1962. doi: 10.3390/jcm12051962 (PMC10004205; doi:10.3390/jcm12051962)
Supplement: Supplementary file 1 [file jcm-12-01962-s001.zip › jcm-2207214-supplementary.pdf]

Journal of Clinical Medicine

MDPI

SUPPLEMENTARY MATERIALS

Table S1. Individual components of the primary endpoint of non-operated AD patients.

	N	Events	PY	Rate (%) [a]
**Composite outcome (primary outcome)**				
Class 0	424	197	1531	12.86
Class 1	676	325	2321	14.00
Class 2	1035	434	3898	11.13
Class 3	1100	397	4329	9.17
Class 4	697	226	2809	8.05
**Rehospitalization associated with aortic dissection**				
Class 0	424	13	1531	0.85
Class 1	676	55	2321	2.37
Class 2	1035	99	3910	2.53
Class 3	1100	138	4332	3.19
Class 4	697	116	2821	4.11
**Referred to aortic surgery**				
Class 0	424	7	1553	0.45
Class 1	676	39	2348	1.66
Class 2	1035	65	3981	1.63
Class 3	1100	99	4426	2.24
Class 4	697	89	2879	3.09
**All-cause death**				
Class 0	424	189	1565	12.08
Class 1	676	290	2452	11.83
Class 2	1035	364	4152	8.77
Class 3	1100	286	4692	6.10
Class 4	697	129	3135	4.11

[a] Rate was calculated as events divided by person-years, presented as %.
